# Evolution of Bionanocomposites: Innovations and Applications in Food Packaging

**DOI:** 10.3390/foods13233787

**Published:** 2024-11-25

**Authors:** Vimala S. K. Bharathi, Digvir S. Jayas

**Affiliations:** 1 Office of the President, University of Lethbridge, 4401 University Drive West, Lethbridge, AB T1K 3M4, Canada; vimalabharathi.sella@uleth.ca; 2 Department of Biosystems Engineering, E2-376 EITC, 75 Chancellors Circle, University of Manitoba, Winnipeg, MB R3T 5V6, Canada

**Keywords:** bionanocomposites, biopolymers, trend analysis, active packaging, antimicrobial

## Abstract

Bionanocomposites are emerging as a pivotal innovation in sustainable food packaging, leveraging the strengths of biopolymers enhanced with nanoparticles for improved functionality. The increasing demand for sustainable packaging solutions, coupled with advancements in nanotechnology, has driven research in this field over the past decade. This review covers the full spectrum of developments in the field, from the classification and synthesis of bionanocomposites to their applications in food packaging and current research trends. A detailed trend analysis using Web of Science data highlighted the growth in bionanocomposite research, with over 17,000 articles published on this topic. Notably, more than 2000 of these articles focus specifically on packaging applications. This review also investigates the application trends for various food products, including fruits and vegetables, grains, meat, dairy products, bakery items, nuts, and oils. The review identifies a marked increase in publications related to bionanocomposite packaging since 2008. Notably, research on packaging applications has increasingly concentrated on fruits and vegetables, followed by meat, dairy products like cheese, and bakery products such as bread. A comprehensive analysis of research trends before 2010 and in 2024 underscores a shift from fundamental material science towards practical, real-world applications. This review provides valuable insights into the transformative potential of bionanocomposites for food packaging technologies and their role in advancing environmentally sustainable solutions.

## 1. Introduction

The emergence of polymers has outsmarted other traditional food packaging materials due to their flexibility, durability, and convenience. However, significant downsides, such as their troublesome disposal, the reduced availability of fossil fuels, and their negative environmental and health impacts, have prompted scientists and industrialists to seek alternatives. While biopolymers have potential, they face challenges due to their inferior barrier and mechanical properties compared to commercial synthetic polymers. Additionally, issues related to their bulk production and cost have hindered their commercial application in the packaging sector.

**Figure 1:**
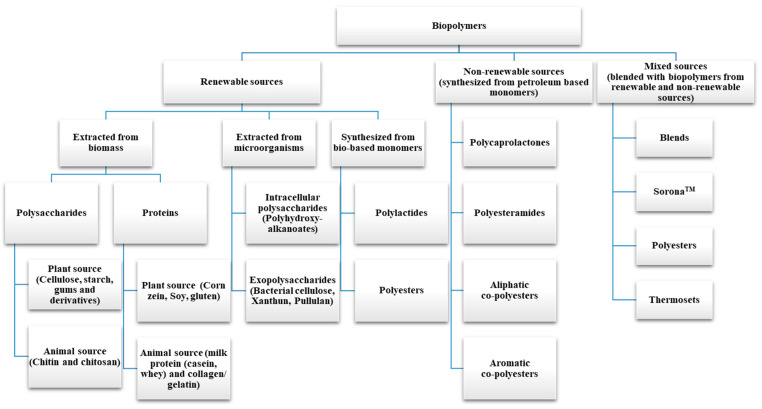
Classification of biopolymers based on source.

The use of biopolymers is now being explored in various fields, including but not limited to automobile, textile, construction, food, biomedical, and packaging. Owing to their increasing popularity and production, researchers from various fields have classified biopolymers in several ways. For instance, based on source and biodegradability, biopolymers can be categorized as bio-based polymers that are biodegradable, bio-based polymers that are non-biodegradable, and petroleum-based polymers that are biodegradable [1]; based on polymeric backbone, biopolymers can be classified into polycarbonates, polyesters, polyamides, polysaccharides, and vinyl polymers; based on the nature of their repeating units, biopolymers can be classed as polysaccharides, proteins, and nucleic acids, with sugars, amino acids, and nucleotides as repeating units, respectively [2]; based on source, biopolymers can be classified as being obtained from biomass or microorganisms, or as being synthesized from bio-derived monomers and petroleum-based products. Figure 1 provides a detailed classification of biopolymers based on source [3,4,5,6,7]. The use of biopolymers as a primary food packaging material remains limited, especially for high-moisture foods, which can alter the properties of the biopolymer. As an alternative, biopolymer blends have been developed, combining biopolymers with other low-cost materials to enhance their properties and reduce the rate of degradation. Further advancements have led to the formation of bionanocomposites by incorporating nano-sized reinforcements, offering improved properties compared to native biopolymers. In general, nanocomposites are polymer composites formed through the incorporation of nano-sized fillers/reinforcements into the polymer matrix using various techniques. Bionanocomposites are advanced biopolymer composites in which biopolymers are reinforced with nano-sized materials, providing unique advantages for sustainable food packaging. Bionanocomposites, also known as green composites, biocomposites, or nanobiocomposites, [8], exhibit superior moisture and gas barrier properties, mechanical strength, fire resistance, thermal properties, optical properties, and biodegradability, enhancing shelf life and reducing environmental impact [9]. The use of nanomaterials in a polymer matrix can result in enhanced properties compared to micromaterials. Otoni et al. [10] prepared nano- and micro-composites, using nano- and micro-droplets of Acetam and Tween 60 as well as nano- and micro-emulsions of carvacrol and cinnamaldehyde, incorporated into isolated soy protein. The study found that the reduced porosity of nanomaterials compared to micromaterials resulted in nanocomposites with lower water vapor permeability. Moreover, the high surface-to-volume ratio of the nano-sized antimicrobial agents boosted the surface reactivity, thereby enhancing the antimicrobial properties [8]. Enhancing the properties of bionanocomposites can facilitate the downgauging of materials, potentially reducing material needs, without compromising function, thus supporting sustainable packaging. This review covers the classification, synthesis, and properties of bionanocomposites, recent advances in food packaging applications, environmental concerns, and an analysis of research trends over the past three decades.

## 2. Classification of Nanocomposites

**Figure 2:**
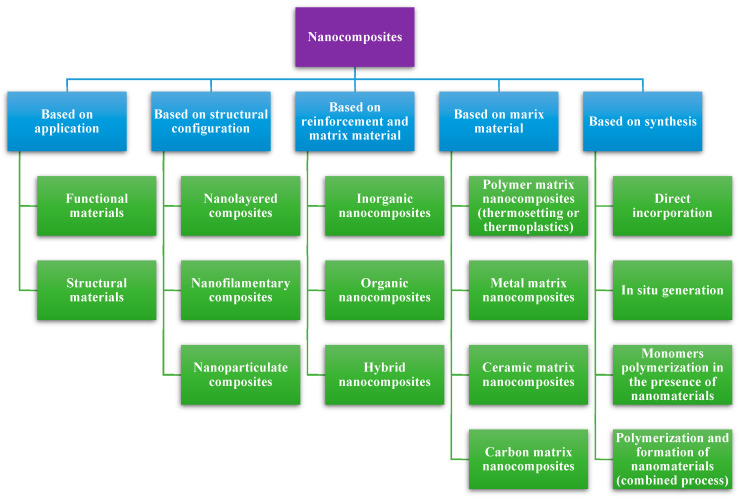
Classification of nanocomposites.

Nanocomposites are classified according to factors like their application, structural configuration, synthesis, reinforcement characteristics, and matrix material (Figure 2) [9,11,12]. Among these, polymer-based nanocomposites are further classified into polymer/ceramic nanocomposites, inorganic/organic polymer nanocomposites, inorganic/organic hybrid nanocomposites, polymer/layered silicate nanocomposites, polymer/polymer nanocomposites, and bionanocomposites. These nanomaterials are widely used in various sectors, including but not limited to automobile, electronics, communication, marine, aerospace, textile, fabrication, construction, pollutants removal, and in biomedical applications [9,13]. However, this review focuses on the bionanocomposites used in food applications.

In bionanocomposites, often, the continuous phase is a biopolymer matrix, and the dispersed phase comprises either inorganic or organic nanoreinforcements. Some of the commonly used nanoreinforcements include clay/layered silicates (montmorillonite (MMT), cloisite (30B, 20A, and 10A), halloysite, kaolinite) biopolymer-based nanoreinforcements (cellulose nanocrystals, chitosan nanoparticles, and nanocellulose isolate from a number of starch sources), and metals and metal oxides like silver, gold, copper, silver oxide, zinc oxide, and magnesium oxide [14]. Among these reinforcements, layered silicates like MMT and kaolinite are preferred for food packaging application, owing to the drastic increase in barrier properties due to the tortuous pathway followed by the permeant, as well as their compatibility, cheap and widespread availability, and high aspect ratio [15,16]. Moreover, these nanoreinforcements are 2D nanomaterials with a 1 nm thickness and length of several µm. In general, the tactoid form is the form in which layered silicates exist in the micro-composites. In these structures, although the layered silicates and the polymer exist in a composite form, they do not properly mix with each other, resulting in the formation of less efficient nanocomposites. However, in the case of nanocomposites, the layered silicates exist either in an intercalated or exfoliated form. In the intercalated form, the multi-layered silicates penetrate the polymer chain, while in the case of the exfoliated form, the layered silicates delaminate into the polymer chain. Because of the availability of layered silicates throughout the nanocomposite in the dispersed form and the enhanced tortuous pathway, exfoliated nanocomposites have been proven to have superior properties [15].

## 3. Synthesis of Nanocomposites

The method of the synthesis of nanocomposites varies with the reinforcement properties, the matrix material used, and the nature of the nanocomposite required. Some of the commonly used techniques for synthesis, like in situ polymerization, melt intercalation, intercalation from solution, emulsion polymerization, melt compounding, sol–gel technology, in situ generation, and electrospinning, are explained in this section.

In situ polymerization encompasses the mixing of monomers in the matrix with layered silicates, thus resulting in the swelling of layered silicates, followed by the polymerization of the monomers, initiated with the help of external stimuli like temperature, radiation, or suitable initiators [17].

Melt intercalation is the process of the amalgamation of the molten matrix polymer and the layered silicates by means of an extruder, ultrasonicator, reflux, or mixer. The level of exfoliation/intercalation of the layered silicate depends on the properties of the matrix polymer and the layered silicates. This technique is considered “green” due to the absence of solvents [18].

Melt compounding is similar to the method of melt intercalation, in which the mixing of molten polymers and layered silicates is achieved via thermomechanical mixing using a screw extruder, compression molding, or injection molding. This technique cannot be adapted to thermally unstable polymers [19].

Intercalation from solution, also known as solution casting, is a three-step process. The first step involves the swelling of the layered silicates through the use of solvents, followed by the addition of a matrix polymer to the solution. Finally, evaporation under vacuum and/or freeze-drying facilitates the removal of the solvent. The solvent can also be removed via precipitation. The desorption of the solvent results in an increase in entropy, and hence the intercalation of polymers and the layered silicates [20].

Emulsion polymerization is the process of the emulsification of monomers (oil/water) in the continuous solvent phase (water/oil) with the help of surfactants. For intercalation to occur, this polymerization process takes place in the presence of layered silicates [18].

Sol–gel technology, also known as in situ template synthesis, is the process of the application of a hydrothermal treatment to a mixture consisting of monomers and precursors of the matrix material and the layered silicates. This results in the formation of nanocomposites through crystallization. The main drawbacks of this technology are the degradation of the matrix material due to the high temperature employed during preparation and the possible aggregation of the nanocomposite that is formed [19].

In situ generation is the process of the generation of nanomaterials using suitable precursors and the reducing agent inside the matrix material (polymer). In some cases, the matrix material itself acts as the reducing agent [12].

Electrospinning is one of the electrohydrodynamic processes, which involves the production of fibers through the application of a high voltage potential to the tip of the needle that is discharging the polymer and the layered silicate solution at a predefined flow rate. The spinning of fibers from the solution occurs mainly due to electrostatic repulsion and the coulombic force acting on the droplet of the solution. Through these forces, the hemispherical-shaped droplets result in the formation of a conical shape (Taylor cone) [21]. Upon the application of a continuous electric potential, the surface tension of the droplet is counteracted by the electrostatic force, leading to jet formation, which is collected by the collector plate connected to the ground.

## 4. Properties of Bionanocomposites

Bionanocomposites often exhibit improved properties compared to pure biopolymer. However, under certain conditions, enhancing one property through the addition of a reinforcement can lead to the decline in another. For instance, adding higher concentrations of natural cellulosic fibers to poly (3-hydroxybutyrate-co-3-hydroxyvalerate) increases the tensile modulus and desorption diffusivity but decreases the solubility of carbon dioxide [22]. Conversely, some reinforcements may not significantly impact certain properties of the biopolymer. For instance, the incorporation of cellulose nanocrystals did not significantly affect the glass transition properties of pectin-based bionanocomposites [23].

The effect of nanoreinforcements on the bionanocomposites varies according to factors such as the concentration [24], type [25], angle of orientation [26], and shape and size/aspect ratio of the nanoreinforcement [27], as well as its affinity/compatibility with the matrix polymer [28], the use of external additives other than the nanoreinforcement in the synthesis [29], and the degree of exfoliation/aggregation. Some of these factors are explored in this section.

### 4.1. Characteristics of the Nanoreinforcement and the Matrix Material

Different nanofillers exhibit varied properties, such as different thermal properties and optical properties, as well as varying compatibility with the matrix material, biodegradability, and toxicity; these play a crucial role in determining the properties of the bionanocomposite. In a study by Ali et al. [30], two types of reinforcements, cellulose crystals and starch crystals, were integrated into a starch matrix material. The resulting bionanocomposites demonstrated distinct advantages. Cellulose crystal-reinforced nanocomposites exhibited enhanced thermal stability, processibility, and mechanical properties, while starch crystal-reinforced composites offered superior UV protection. Thus, the properties of the bionanocomposites can either improve or decline depending on the nature of the reinforcements used.

Similar reinforcement materials synthesized from different sources can have significantly different effects on the bionanocomposite. For instance, the incorporation of cellulose nanocrystals obtained via acid hydrolysis, using acids such as sulfuric, phosphoric, hydrochloric, and nitric acid, into poly(lactic acid) exhibited diverse properties [31] Cloisite Na^+^ (natural form) and Cloisite 10A (organically modified form)-reinforced sesame seed meal protein composite films showed significantly different properties [25]. Chatterjee et al. [27] explored the effect of graphene nanoplatelets with two different flake sizes (5 and 25 µm) on epoxy-based nanocomposites and found that nanocomposites reinforced with 25 µm graphene nanoplatelets showed superior properties. Moreover, the study also evaluated the effect of mixtures of carbon nanotubes and graphene nanoplatelets and proved that the addition of hybrid reinforcements into the polymer matrix can have a synergistic effect on the nanocomposite. Similarly, Makwana et al. [32] prepared a silver-modified MMT-reinforced agar–carboxymethyl cellulose bionanocomposite and observed the synergistic effect in terms of the antimicrobial activity of silver and the enhanced mechanical and thermal properties of MMT. A similar study compared the effects of MMT–copper oxide (CuO) nanoparticle reinforcement compared to chitosan with only MMT reinforcement at various concentrations, such as 1, 3, and 5 wt%, and found that MMT-CuO exhibited superior mechanical, moisture, and gas barrier properties, as well as superior antimicrobial properties and reduced water solubility, compared to the corresponding MMT-reinforced bionanocomposites [33].

Alternatively, the combined use of different matrix materials can also improve the properties of bionanocomposites. Marvizadeh et al. [34] proved that a nanorod zinc oxide-reinforced starch–gelatin bionanocomposite had superior properties compared to starch or gelatin nanocomposites. On the other hand, the interaction between the matrix material and nanofiller depends on the type of matrix used. Therefore, the same nanofiller can affect different polymers in various ways. For instance, copper oxide nanoparticles were incorporated into several carbohydrate biopolymers, including agar, alginate, carrageenan, chitosan, and carboxymethyl cellulose (CMC). All biopolymers, except chitosan, were dissolved in double-distilled water, while chitosan was dissolved in a 1% (*v*/*v*) acetic acid solution. A consistent procedure was used to create bionanocomposites with 2% (*w*/*w*) nanofiller in each dry biopolymer. Despite the same amount of each antimicrobial agent being used, the antimicrobial activity of the nanocomposite films against *Listeria monocytogenes* and *Escherichia coli* varied. Agglomeration due to the affinity of the matrix polymer to the reinforcement material limited the release of copper ions from the bionanocomposite films. Moreover, the tensile strength of the bionanocomposites improved significantly with the addition of reinforcement materials compared to the pure biopolymers, obtaining the following results: agar (9.35 times), alginate (27.15 times), carrageenan (2.59 times), chitosan (46.54 times), and CMC (18.64 times) [28]. Similarly, when comparing the mechanical properties of MMT nanoplatelets, a bamboo nanofiber-reinforced chitosan, and a cassava starch bionanocomposite, the cassava starch-based bionanocomposite showed an increase in tensile strength when both nanoreinforcements were used, while chitosan-based bionanocomposite showed a decrease in tensile strength. This variation is attributed to the interaction between the polymer matrix and the nanomaterials [35]. Additionally, the amylose contents in starch were also found to influence the characteristics of the nanocomposite-forming solution, as well as the nanocomposite [36]. This highlights the significant role of the matrix material in determining the reinforcement’s impact on the nanocomposite properties.

### 4.2. Arrangement of Nanoreinforcements in Nanocomposites

Reinforcements with a high aspect ratio, when arranged in such a way that their larger surfaces are perpendicular to the permeant molecules, often obtain the best barrier properties. For spherical and cubical particle-reinforcements, the effect of arrangement on reinforcement is minimal. The arrangement of the nanoreinforcements mainly depends on the method of synthesis. For instance, solution intercalation and in-situ polymerization are more effective in producing properly arranged nanoreinforcement-based nanocomposites compared to melt intercalation [37]. A bionanocomposite was prepared using Jamaica (*Hibiscus sabdariffa)* flower extract nanopacked into natural and modified MMT clays, which were then reinforced in thermoplastic corn starch. The nanopacking of the extract increased the interlayer spacing of the nanoclays and improved their exfoliation, leading to a better interaction with the starch matrix. However, modified MMT-reinforced nanocomposites exhibited superior moisture barrier properties compared to those using a nanopacked extract. The parallel orientation of the nanoreinforcements affected water vapor permeability as well as opacity. Also, starch retrogradation was found to correlate with orientation, thus affecting the interaction of the matrix material with the reinforcement [26]. These studies help us to understand that the arrangement of nanoreinforcements within the polymer matrix influences the final properties of the bionanocomposites.

### 4.3. Concentration

The concentration of the nanoreinforcement that is added plays a significant role in determining the properties of the bionanocomposites [38]. The addition of a significantly lower concentration of reinforcements can often lead to drastic improvement in the properties of the polymer. For instance, the addition of a very low concentration (0.04 *w*/*w*%) of silver nanoparticles to poly (3-hydroxybutyrate-co-3-hydroxyvalerate) improved the oxygen barrier property by almost 56% compared to that of pure polymer [39]. In general, with an increase in the concentration of the nanoreinforcement, the effect on the bionanocomposite also increases. However, a threshold value exists, above which the effect on the bionanocomposite reduces. For example, Sahraee et al. [24] prepared gelatin-based bionanocomposite reinforced with chitin nanoparticles (0, 3, 5, and 10% (*w*/*w* of dry gelatin)). Upon analyzing the antifungal activity of the composite against *Aspergillus niger*, 5% showed a higher diameter of inhibition (15.72 mm), whereas 10% showed a lower diameter of inhibition (4.78 mm) than the diameter obtained by the 3% composite (6.56 mm). A similar trend was observed in terms of mechanical properties as well [24]. This could be attributed to the agglomeration of nanoreinforcements, resulting in non-uniform dispersion, which could create a void space in the nanocomposite that reduces the effect on the properties of the bionanocomposite. This concept does not affect properties such as the thermal property. Hence, those properties exhibit a uniform trend. Table 1 explains the optimum concentration/threshold value of reinforcements based on the analyzed properties of the nanocomposites. In a few cases, the concentration of the nanoreinforcement may not have significant effect on the properties of the composites. Increasing the concentration of an MMT (Cloisite 30B) did not have an effect on the thermal properties of a poly(vinyl alcohol) (PVA)–chitosan-based nanocomposite [40]. In contrast, the thermal stability of a nano-calcium–carbonate-reinforced starch bionanocomposite significantly improved with an increase in the concentration of the reinforcement [41].

**Table 1:** Optimal nanoreinforcement concentration for bionanocomposites.

Matrix Material	Reinforcement	Concentrations Tested (wt%)	Optimal Concentration (wt%)	Analyzed Properties	References
Potato starch	Cellulose nanofibres	0, 1, 2, 3, 4	3	Contact angle, water sorption, and moisture barrier properties	[42]
Pectin	Cellulose nanocrystals	0, 2, 5, 7	5	Mechanical and moisture barrier properties	[23]
Sugar palm starch	Sugar palm nanocrystalline cellulose	0, 0.1, 0.2, 0.3, 0.4, 0.5, 1	0.5	Mechanical properties	[43]
Sesame seed meal protein	Cloisite Na^+^	0, 1, 3, 5, 7	5	Mechanical and moisture barrier properties	[25]
Agar-Carboxymethyl cellulose	Silver-modified montmorillonite	0, 2, 3, 5, 8	5	Mechanical properties	[32]
Chitosan	Montmorillonite –copper oxide	0, 1, 3, 5	3	Mechanical, moisture and gas barrier, and antimicrobial properties	[33]
Poly (lactic acid)	Halloysite clay	0, 2, 4, 6, 8	2	Mechanical properties	[44]
Gelatin	Chitin nanoparticles	0, 3, 5, 10	5	Mechanical and antimicrobial properties	[24]
Soybean polysaccharide	Montmorillonite	0, 5, 10, 15	10	Mechanical properties	[45]
Agar	Silver nanoparticles	0.5, 1, 1.5, 2	1	Mechanical and moisture barrier properties	[46]
Chitosan	Titanium dioxide	0, 0.25, 0.5, 1, 2	1	Mechanical, moisture barrier, and ethylene scavenging activity	[47]

### 4.4. Method of Synthesis

A slight modification to the method of the synthesis of nanocomposites can have a substantial impact on the final properties. To increase the level of exfoliation of the layered silicates, and hence the performance of the nanocomposites, mechanical treatments like ball-milling, ultrasonication, shear mixing, and thermal expansion are often carried out. Zhou et al. [48] claimed that the organic modification of MMT before its incorporation into oxidized starch can expand the interlayer spacing, and hydrophilic ball-milling can exfoliate the MMT clay. They showed that a modified MMT (5 wt%)-reinforced starch nanocomposite exhibited a substantially higher breaking strength, percentage elongation, fracture work, and initial modulus than native starch. A study conducted to analyze the effect of ultrasonic vibration time on the mechanical properties and crystallinity index of yam bean thermoplastic starch reinforced with water hyacinth nanofiber cellulose (1 wt%) proved that ultrasonic vibration can enhance the properties drastically, reducing the amount of reinforcement that needs to be used [49]. During the preparation of a TiO_2_-reinforced starch bionanocomposite, Goudarzi and Shahabi-Ghahfarrokhi [50], exposed the nanocomposite film-forming solution to UV-A lamps for 1, 6, and 12 h prior to drying, and found that UV-A exposure time has a noteworthy negative impact on tensile strength and Young’s modulus. Moreover, properties like elongation at break, glass transition temperature, and the melting point of the films were found to increase with UV-A exposure time.

Romero-Bastida et al. [36] prepared corn starch–MMT-based nanocomposites using glycerol as the plasticizer. During the preparation, similar procedures were followed, except the order in which components were added. Briefly, in method 1, starch and glycerol were added prior to the addition of the reinforcement, while in method 2, the plasticizer (glycerol) was added after the addition of the reinforcement to the starch. A better starch–MMT interaction was obtained in the nanocomposites obtained using method 2, which had superior mechanical properties compared to the bionanocomposite [36]. Similarly, two different starch–MMT bionanocomposites were prepared, with a slight difference in their preparation method. In one method, the starch, glycerol (plasticizer), and the reinforcement materials were mixed in one step; in the second method, the reinforcement material was diluted with water to enhance the clay-opening and facilitate a better interaction between the starch polymeric chain and the interfacial layers of the nanoclay. The nanocomposites prepared with the latter method showed a reduction in water vapor permeability, water uptake, opacity, and solubility at all concentrations (1, 3, 5, 7, 10 wt%) compared to the corresponding nanocomposites prepared using the former method [51].

### 4.5. Addition of External Additives

External additives interact with the matrix material and the reinforcement, leading to modifications to the properties of the nanocomposites. Gutiérrez and Alvarez [52] prepared nanocomposites using thermoplastic corn starch as the matrix material and modified MMT as the nanoreinforcement; glycerol was added as the plasticizer. The study evaluated the effect of the incorporation of a blueberry (*Vaccinium corymbosum)* extract nano-packed within the nano-clays on the properties of bionanocomposites and found that the average roughness, Young’s modulus, and maximum stress values of nanocomposites containing extract nano-packed reinforcements were higher than those of their corresponding nanocomposites without the extract. On the other hand, another study evaluated the effect of citric acid, a crosslinker, on the properties of a cellulose nanocrystal-reinforced pectin bionanocomposite and found that the addition of a crosslinker enhanced the tensile, moisture barrier, opacity, and water-resistance properties of the bionanocomposite, further enhancing the stiffness of the polymer chains [53]. Upon evaluating the effect of various additives, like glycerol, citric acid, and ascorbic acid, on the nano-zinc sulfide-containing mungbean starch/PVA bionanocomposites, Yun et al. [54] revealed that all three additives led to significantly different swelling degrees, solubilities, and mechanical properties at various concentrations (0.5, 0.8, 1.2, 1.5, 2.0 wt%).

The addition of external agents like plasticizers, antimicrobial agents, or antioxidants can negatively impact other properties, such as barrier or mechanical properties. Risyon et al. [44] found that the addition of glycerol as a plasticizer prevented the penetration of the polymer matrix into the reinforcement through intercalation with the reinforcement material, thus reducing the mechanical properties of the nanocomposite. The addition of potassium sorbate as an antimicrobial agent for starch–clay bionanocomposites served as a potential active packaging material against *A. niger*. However, with an increase in the concentration of potassium sorbate, the tensile strength decreased, and water permeability and elongation at break increased [55]. Hence, the compatibility of the additives with the bionanocomposites must be taken into consideration. In some cases, the additives are used as coatings in the reinforcement material or the bionanocomposite itself, to enhance their properties. Oymaci and Altinkaya [56] coated zein nanoparticles with sodium caseinate to ensure the uniform distribution of reinforcements in the whey protein isolate bionanocomposites. This was due to the presence of both hydrophilic and hydrophobic groups of sodium caseinate, which ensures the uniform distribution of reinforcement through interactions with the hydrophobic groups of zein nanoparticles and hydrophilic groups of whey protein isolate. Thus, the addition of a sodium caseinate coating to the hydrophobic reinforcement material helped ensure a homogeneous distribution even when using a higher concentration of the reinforcement [56].

### 4.6. External Factors

The properties of the bionanocomposite are also influenced by external environments such as temperature, pH, moisture, and exposure to sunlight. These parameters must be taken into account, especially when bionanocomposites are applied as the primary packaging for food packaging application. A study compared the equilibrium swelling ratio of bionanocomposite hydrogels reinforced with cellulose nanocrystals into xanthan gum solutions and chitosan at various pHs (2, 4, 5, 6.5, 7, 7.4 and 10) for 3 days. The equilibrium swelling ratio of the bionanocomposites with reinforcement concentrations of 0, 2, 5, and 10% showed a similar trend, with the minimum water uptake of the polyelectrolyte occurring at pHs 5 and 6.5 and the maximum uptake occurring at 7.4 and 10. A lower swelling ratio within the pH range from 3 to 6.5 was observed; this could be attributed to the rigid network structure formed by chitosan and xanthan at that particular pH range [57]. Starch bionanocomposites with added titanium dioxide were found to degrade upon UV-A exposure [50]. Likewise, the exposure of cellulose nanofiber-reinforced starch bionanocomposites to microwave energy led substantial variations in the bionanocomposites’ properties [58]. These material properties are a major concern when considering the use of bionanocomposites in food packaging applications, especially for foodstuffs for which microwave energy might be used for cooking, heating, re-heating, and thawing.

### 4.7. Prediction Studies

Considering that the barrier properties are the most significant properties for bionanocomposites used in food packaging applications, research works related to the prediction of nanocomposites’ barrier properties are discussed. Factors like orientation, the degree of exfoliation, aspect ratio, and platelet–platelet overlap are taken into consideration when predicting the barrier properties of bionanocomposites. Initially, Barrer and Petropoulos [59] and Nielsen [60] paved the way for nanocomposite modeling by developing diffusion theories. Nielsen’s model is based on the increase in the path length of the diffusing molecule with respect to the aspect ratio and concentration/volume fraction. This pathway, followed by the permeant molecule, is termed the tortuosity pathway. The theory considers the effect on the permeability of liquid, which acts as a permeant due to the polymer–filler interface and the solubility of the polymer. Moreover, the theory also explains the perpendicular and parallel orientation of the nanoreinforcement [60].

Later, the Nielsen model was modified by considering orientation and alignment, which led to the conclusion that longer exfoliated sheets can enhance the barrier properties of the composite materials [61]. Fredrickson and Bicerano [62] developed a model to predict the barrier properties of randomly oriented disk-shaped reinforcements with a high aspect ratio. This is of considerable interest due to the random orientation of the reinforcements in nanocomposites that are prepared experimentally. Cussler et al. [63] explained the reduction in the barrier properties of the nanocomposites after the addition of nanoreinforcements through experimental studies. According to their model, shape, array, and monodispersity/polydispersity also contribute to the barrier properties of the nanocomposite film. On the other hand, Gusev and Lusti [64] developed models based on computer simulations and mainly considered two factors, specifically, the geometric factor and molecular transformation in the polymer matrix due to the presence of a reinforcement.

Sun et al. [65] studied the effect of nanoplatelet exfoliation and the aspect ratio on the barrier properties of epoxy nanocomposites and compared their results with those of the models presented above. They found that their experimental results correlated with Gusev-Lusti’s model and Nielsen’s model. Minelli et al. [66] confirmed that Nielsen’s model has the potential to make accurate predictions by comparing the mass transport simulations with 3D geometries with different shapes (square, circular, octagonal and tape-shaped), dispersions (random and ordered), volume fractions (2.5, 5, and 10%), aspect ratios (20, 50, 75, and 100), and flake spacings in an ordered structure using a finite volume method-based numerical algorithm. Moreover, the study also proposed that simulations using 2D structures overpredict the effect of reinforcements by exhibiting lower barrier properties for 3D structures than those of 2D structures [66]. However, Nielsen’s tortuous path model failed to explain the reduction in barrier properties observed in practical conditions. Hence, Beall [67] proposed a model called the “conceptual model”, which provides a correction factor to Nielsen’s model, considering the polymer region of 50–100 nm around the nanoreinforcement, called the constrained polymer region. Later, Adame and Beall [68] found a way to measure the size, shape, and diffusion coefficient of the constrained polymer region, which helps in practical implementations of the model.

## 5. Trends and Advances in Bionanocomposites Research

**Figure 3:**
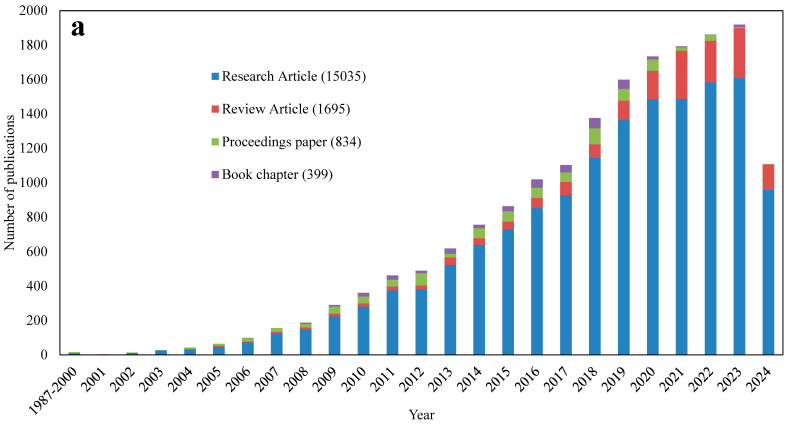

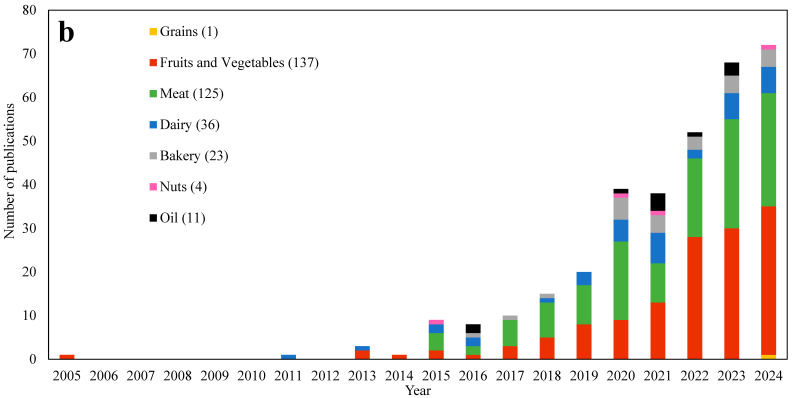
Publication trends in (**a**) bionanocomposites and (**b**) their application in various food products. (Data source: Web of Science.) (The numbers in the parentheses represent the total number of research articles obtained for a particular category).

To understand the evolving trends in bionanocomposites research, with a particular focus on food packaging applications, a detailed analysis was performed using the Web of Science database during the first week of September 2024. This analysis began with a comprehensive keyword search, incorporating terms such as “bionanocomposite”, “nanobiocomposite”, and “biodegradable nanocomposite” in the various forms in which they were used in the literature, including hyphenated, spaced, and plural variations. Publications were categorized into research articles, review articles, conference proceedings, and book chapters to map the overall trend in the field. The data revealed a notable increase in both the volume and diversity of publications over time (Figure 3a).

To compare current research trends on bionanocomposites with those from two decades ago, keywords were extracted from 959 research articles published in 2024 and 976 articles published from 1987 to 2010. The comparison of bionanocomposite research before 2010 and research in 2024 reveals a clear shift in focus from fundamental material science to applied, real-world solutions. Prior to 2010, the research was centered around basic materials such as carbon nanotubes, cellulose, polymers, and starch, with an emphasis on mechanical, thermal, and crystallization properties. In contrast, by 2024, the focus shifted towards practical applications, particularly in food packaging, with keywords such as packaging, antibacterial, and biodegradable becoming prominent. This indicates a growing interest in addressing contemporary challenges such as food safety and environmental sustainability. Additionally, newer, high-performance materials like graphene oxide nanoparticles emerged, showing a move toward more advanced and multifunctional nanomaterials. The research evolved to incorporate not just a fundamental understanding of bionanocomposites but also their application in the development of eco-friendly, antimicrobial, and sustainable packaging solutions. Further analysis with a targeted search combining bionanocomposite-related keywords with “packaging” or “coating” identified 2102 articles dedicated to packaging and coating applications. These articles encompass both fundamental research and studies examining the impact of bionanocomposites on the quality of various food materials. Although research into bionanocomposites began in the late 20th century, the focus on their application in food packaging is a relatively recent development. Notably, a significant increase in research on this topic has only been observed since 2008. This suggests that while the foundational science behind bionanocomposites has been established for several decades, their use in food packaging has only recently gained traction and become a focus of intensive study.

In addition, a focused search of research articles specifically testing the effect of bionanocomposites on the quality of food products such as grains, fruits and vegetables, meat, dairy products, bakery items, nuts, and oils highlights current trends. The primary application of bionanocomposite-based packaging is in fruits and vegetables, followed by meat, dairy products, and bakery items (Figure 3b). This focus on using bionanocomposites for fruits and vegetables, meat, and dairy products is largely due to the need for packaging with effective antimicrobial, ethylene scavenging, and oxygen-scavenging properties for these products as these perishable items are highly sensitive to spoilage. However, future research on bionanocomposites should also prioritize grains, such as canola and flax, which are particularly susceptible to spoilage. Bionanocomposites can play a key role in providing insect-repellent and antifungal properties, making them valuable for enhancing grain preservation and storage.

## 6. Application of Bionanocomposites in Food Packaging

Bionanocomposites are increasingly preferred over polymer nanocomposites for food packaging due to their compatibility with food-grade standards and the GRAS (Generally Regarded as Safe) designation of some biopolymers. However, challenges such as the migration of nanomaterials into food and the hydrophilic nature of these materials need to be addressed. Migration can be minimized using 2D nanomaterials like layered silicates and biobased nanoreinforcements. To reduce their hydrophilic nature, suitable nanoreinforcements and external agents can be employed.

Key attributes for food packaging include excellent barrier and mechanical properties. Superior barrier properties prevent spoilage and extend shelf life, while enhanced mechanical properties protect the food during handling and storage. Since the major studies conducted on bionanocomposites have focused mainly on these two areas, bionanocomposites have been shown to hold significant potential for food packaging applications. Depending on the properties of the nanomaterials used, the bionanocomposites can also provide additional functionalities, such as active and intelligent packaging, offering further improvements in food preservation and monitoring.

### 6.1. Active Packaging

Active packaging describes packaging materials that are specifically designed to extend the shelf life of food or enhance its condition. Active packaging systems can include releasing systems or scavenging systems; the former includes emitters that emit active components like antimicrobial agents, antioxidants, ethylene, or carbon dioxide into the packaged food or headspace of the food container, while the latter involves the presence of absorbers like oxygen, carbon dioxide, ethylene, and moisture scavengers. At first, these scavengers and absorbers were packed into a sachet before being added to the food packaging system. Due to the shortcomings of sachets, like their unsuitability for the packaging of certain high-moisture foods and beverages, and the accidental ingestion of the components by consumers due to damaged sachets, researchers have focused on the preparation of nanocomposites with an active material as a reinforcement or as an external agent in addition to a reinforcement [69]. By reducing the size of the active agent, their reaction rate increases; furthermore, some agents, like gold, are active only at the nano-size.

#### 6.1.1. Antimicrobial Applications

Microbial spoilage is the foremost reason for the spoilage and loss of food materials, especially high-moisture foods [70]. Initially, microorganisms were prevented from spoiling using techniques like heat, drying, salting, and fermentation. As the food industry expanded, chemical preservatives became more common. However, concerns about the health effects of these preservatives led to regulations limiting their use. The increasing consumer demand for preservative-free products has driven research into methods that minimize or eliminate preservatives while maintaining food quality and shelf life. Otoni et al. [71] demonstrated that the incorporation of antimicrobial agents into packaging material can extend the shelf life of sliced bread more effectively than commercial antifungal agents. Moreover, nano-sized antimicrobial agents are more effective than their micro-sized counterparts.

Bionanocomposites can serve as antimicrobial food packaging materials due to the properties of their matrix or reinforcement materials. Matrix materials like chitosan have inherent antimicrobial characteristics. However, the use of antimicrobial agents as reinforcements is more effective owing to their high surface-to-volume ratio. Reinforcements like silver, titanium, zinc, gold, and copper nanoparticles are most commonly used. Owing to their high-thermal stability, broad spectrum of antimicrobial properties, and better activity against resilient microorganisms, silver nanoparticles from various sources have been evaluated for their antimicrobial activity [72]. Moreover, silver nanoparticles outperform other, previously mentioned inorganic antimicrobial agents [73]. A study on the effect of silver nanoparticles’ size and shape on their antimicrobial activity against *Streptococcus mutans* found that neither size nor shape significantly affected the antimicrobial activity [74].

Certain biopolymers with antimicrobial properties, when combined with other antimicrobial reinforcement agents, have an synergistic antimicrobial effect. For instance, chitosan-stabilized silver nanoparticles showed better antimicrobial properties than pure chitosan and silver aqueous suspensions [75]. Similarly, Han et al. [76] found that a chitosan–MMT bionanocomposite showed better antimicrobial activity against *Staphylococcus aureus* and *E. coli* compared to the activity of pure chitosan and MMT. Table 2 illustrates some of the bionanocomposites and their antimicrobial characteristics.

#### 6.1.2. Oxygen Scavenging Systems

Oxidation is another significant cause of food spoilage, leading to off-flavors, rancidity, discoloration, reduced nutritional value, and microbial growth. To combat this, food-grade antioxidants like BHA (Butylated Hydroxy Anisole) and BHT (Butylated Hydroxy Toluene) are used. Techniques such as vacuum- and modified atmospheric packaging help, but their effectiveness is limited by the packaging permeability and the unsuitability of these techniques for foods such as bread, which can collapse under vacuum. Moreover, for porous foods, complete atmospheric modification is challenging. Gutiérrez et al. [77] compared the use of a modified atmospheric packaging and active packaging of gluten-free sliced bread and reported that the active packaging outperformed the modified atmosphere packaging, better preserving the bread’s sensory qualities and inhibiting microbial growth.

Oxygen scavengers offer an effective alternative. These materials react with oxygen and make it unavailable for further oxidation. Combining these potential scavengers with low-permeability packaging can reduce oxygen levels to as low as 0.0001%. The period for which this effect is extended depends on the characteristics of the packaging material [78,79]. The oxygen-scavenging activity of some components needs to be triggered with external stimuli like heat and moisture. For instance, the incorporation of α-tocopherol nanoparticles and iron chloride into a warm-water fish gelatin nanocomposite exhibited a scavenging capacity when triggered with moisture [80]. Similarly, α-tocopherol nanoparticles and iron chloride-incorporated polycaprolactone required heat to exhibit oxygen-scavenging activities [81]. On the other hand, some scavengers, like TiO_2_, require continuous UV light illumination to scavenge oxygen molecules [69].

One study compared the oxygen-scavenging capabilities of electrospun fibers and films of poly(3-hydroxybutyrate) reinforced with palladium nanoparticles. Here, palladium proved to be an effective oxygen scavenger. The electrospun fibers containing nanoparticles showed a significantly higher oxygen-scavenging activity compared to films. This increase in scavenging activity is due to the fact that the fibers possess a high surface-to-volume ratio and high porosity, which makes the palladium nanoparticles are able to scavenge oxygen. Moreover, the study also found that the higher RH facilitates an increase in scavenging activity. Thus, the developed electrospun fibers can be applied as an inner layer in active food packaging applications [82].

While the current research on bionanocomposites with oxygen-scavenging systems shows promising results in terms of enhancing food preservations and extending shelf life, future studies should focus on optimizing the performance of these systems across various food types and packaging conditions. Continued innovation and exploration in this field will be crucial for developing more efficient, sustainable, and cost-effective food packaging technologies.

#### 6.1.3. Ethylene Scavenging Systems

Ethylene, a hormone released by climacteric fruits, accelerates ripening and leads to spoilage, including tissue softening and deterioration. To address this issue, ethylene scavengers are employed. These scavengers work by either oxidizing ethylene into carbon dioxide and water or sorbing the ethylene.

**Table 2:** Bionanocomposites and their antimicrobial characteristics.

Matrix	Reinforcements and/or Additives	Antimicrobial Agent	Concentration	Microorganisms Tested *	Research Findings	References
Guar gum	Silver–copper alloy nanoparticles	Reinforcement	0.5, 1, 2 wt%	*L. monocytogenes*, *S. typhimurium*	Antimicrobial activity increased with an increase in reinforcement concentration. *Salmonella typhimurium* was more susceptible compared to *L. monocytogenes*.	[83]
Bovine skin gelatin	Zinc oxide nanorods; clove essential oil	Reinforcement and antimicrobial additive	Zinc oxide—2 wt% gelatin; essential oil—25 and 50% wt of protein content	*L. monocytogenes*, *S. typhimurium*	Maximum microbial inactivation at higher essential oil concentrations. Complete inactivation of microorganisms after 7 days of incubation.	[84]
Chitosan	Nanosized titanium dioxide	Matrix material and reinforcement	1 wt%	*E. coli*, *S. typhimurium*, *P.aeruginosa*, *A. oryzae*, *P. roqueforti*	Synergistic antimicrobial activity against the tested microorganisms due to photocatalytic activity of titanium dioxide and antimicrobial properties of chitosan.	[85]
Starch	Halloysite; nisin	Antimicrobial additive	Halloysite—3, 6 wt%; Nisin—2, 6 wt%	*L.monocytogenes*, *S. aureus*, *C. perfringens*	Nisin added nanocomposite exhibited the highest antimicrobial activity against *C. perfringens*, followed by *L. monocytogenes*.Antimicrobial activity increased with an increase in nisin concentration and decrease in nanoclay concentration.	[86]
Agar/banana powder	Silver nanoparticles	Reinforcement	1 mM silver; 4/0, 3/1, 2/2, 1/3, and 0/4—blending rations of agar and banana powder	*E. coli*, *L. monocytogenes*	Increase in antimicrobial activity with an increase in banana powder.Only bacteriostatic activity against *L. monocytogenes*.Bactericidal and bacteriostatic activity against *E. coli* were observed at higher and lower concentrations of banana powder.	[87]
Cellulose Acetate Butyrate	Cloisite 30B; carvacrol and cinnamaldehyde	Reinforcement and antimicrobial additive	Cloisite 30B—5 wt%; carvacrol—10 wt%; cinnamaldehyde—10 wt%	*L. innocua*, *E. coli*, *S. cerevisiae*, *S. aureus*	Antimicrobial activity against *S. cerevisiae* with the addition of Cloisite 30B. Higher log reduction in cinnamaldehyde-incorporated films compared to carvacrol.	[88]
Chitosan	Zinc oxide nanoparticles; Neem essential oil	Matrix, reinforcement, and antimicrobial additive	Zinc oxide nanoparticles—0.1, 0.3, and 0.5%	*E. coli*	Increase in antimicrobial activity with increase in nanoparticle concentration.	[89]
Agar	Silver nanoparticles	Reinforcement	0.5, 1, 1.5, 2 wt%	*E. coli*, *L. monocytogenes*	Higher antimicrobial activity against *E. coli* compared to *L. monocytogenes*. Increase in antimicrobial activity with increase in nanoparticle concentration.	[46]
Chitosan/ Carboxymethyl cellulose	Zinc oxide nanoparticles	Reinforcement	2, 4, 8 wt%	*S. aureus*, *P. aeruginosa*, *B. cereus*, *B. subtilus*, *L. monocytogenes*, *E. coli*, *C. albicans*, *A. niger*	Antimicrobial activity against all the microorganisms tested. Higher zone of inhibition (range: 5 to 15 nm) for *B. cereus* and *S. aureus. S. aureus.*	[90]
Chitosan/ guar gum	Zinc oxide nanoparticles (prepared with roselle calyx extract)	Reinforcement	1, 3 wt%	*B. cereus*, *L. monocytogenes*, *A. flavus*, *E. coli*, *S. typhimurium*, *A. niger*, *S. aureus*, *Y. enterocolitica*, *A. terries*, *P. aeruginosa*	Higher nanocomposite activity than reselle calyx coating.Higher inhibition zone for *E. coli*, followed by *L. monocytogenes*, and *A. terries*.Lowest inhibition for *P. aeruginosa*.	[91]

* *A. flavus*—*Aspergillus flavus; A. niger*—*Aspergillus niger; A. oryzae*—*Aspergillus oryzae; A. terries*—*Aspergillus terries; B. cereus*—*Bacillus cereus; B. subtilus*—*Bacillus subtilus; C. albicans*—*Candida albicans; C. perfringens*—*Clostridium perfringens; E. coli*—*Escherichia coli; L. innocua*—*Listeria innocua; L. monocytogenes*—*Listeria monocytogenes; P. aeruginosa*—*Pseudomonas aeruginosa; P. roquefort*i—*Penicillium roqueforti; S. aureus*—*Staphylococcus aureus; S. cerevisiae*—*Saccharomyces cerevisiae; S. enteri*c—*Salmonella enteric; S. typhimurium*—*Salmonella typhimurium; Y. enterocolitica*—*Yersinia enterocolitica*.

While traditional scavengers like potassium permanganate and activated charcoal have been used as oxygen scavengers, naturally occurring materials such as cloisite, halloysite, and MMT are gaining attention due to their ethylene scavenging properties [92]. Recent studies highlight the effectiveness of halloysite nanotubes, which possess a hollow structure and large aspect ratio, in adsorbing ethylene. Tas et al. [93] demonstrated that these nanotubes effectively reduced ripeness and firmness issues in bananas and tomatoes. The nanotubes also improved the oxygen and moisture barrier properties, extending the shelf life of fresh-cut chicken and strawberries. Further research by Gaikwad et al. [92] showed that alkaline-treated halloysite nanotubes have enhanced ethylene scavenging capabilities due to their larger pore size. Joung et al. [94] incorporated halloysite nanotubes containing potassium permanganate into low-density polyethylene and showed that the prepared nanocomposite could reduce the ethylene production and respiration rate of cherry tomatoes, in addition to delaying the decrease in firmness and color changes.

Cellulose nanofiber-based films combined with TiO_2_ nanotubes (modified by Cu_2_O) effectively scavenged the ethylene produced in the headspace during the storage of packaged tomatoes, delaying discoloration, softening, and weight loss [95]. Similarly, corn starch and gum acacia films combined with sepiolite demonstrated effective ethylene-scavenging for broccoli florets packaged at 23 °C for 6 days, with increased scavenging properties at higher sepiolite concentrations [96]. Fan et al. [97] developed a paper towel microfiber-based film combined with core–shell-structured zein-*Artemisia sphaerocephala* Krasch. gum nanoparticles. Their study demonstrated that bananas packaged with these films exhibited reduced browning, increased hardness, and an extended shelf life compared to untreated bananas. Bionanocomposites hold significant promise for ethylene-scavenging applications, offering innovative and sustainable solutions to mitigate the post-harvest spoilage of fruits and vegetables.

#### 6.1.4. Multiple Active Applications

The matrix material and reinforcement selection in bionanocomposite films can enable multiple active functions, enhancing the shelf life of packed food. For instance, TiO_2_-reinforced chitosan bionanocomposites demonstrate both ethylene scavenging as well as antimicrobial activities. Specifically, the developed bionanocomposite increased the photodegradation of ethylene with the addition of higher concentrations of TiO_2_ and showed antimicrobial activity against *S. aureus*, *E. coli*, *Salmonella typhimurium*, *Pseudomonas aeruginosa*, *Aspergillus*, and *Penicillium* [47]. Fernández et al. [98] developed an absorbent pad based on cellulose fibers and a silver nitrate composite and studied its effect on a minimally processed “Piel de Sapo” melon stored at 4 °C for 10 days. The moisture from the melon triggered the release of silver nanoparticles from the pad. These nanoparticles exhibited a strong antimicrobial effect, particularly on yeast, followed by aerobic mesophilic and psychrotropic microorganisms. Also, the silver nanoparticles were found to have an effect on ethylene as well, thus resulting in reduced ripening rate, as is evident from the decreased °brix values and the visual appearance of the melon samples. Unlike traditional packaging, bionanocomposites have the ability to integrate multiple active applications within a single material. This versatility makes bionanocomposites a more efficient and sustainable option for food preservation, as they reduce the need for multiple packaging layers or additives, streamlining the packaging process.

### 6.2. Intelligent Packaging

Intelligent packaging, also known as smart packaging, refers to packaging systems designed to monitor the conditions of packaged food to provide real-time information on the quality and safety of the packaged food material. These systems include elements such as time–temperature indicators, freshness indicators, Radio-Frequency Identification (RFID) tags, and barcodes. With the increasing demand for traceability, transparency, and food safety, intelligent packaging systems are gaining attention in food applications.

#### 6.2.1. Time–Temperature Indicators

Time–Temperature indicators (TTI) serve as a visual tool for assessing food quality by displaying color changes in response to temperature exposure over time. By monitoring both the duration and intensity of the temperature fluctuations, TTIs help predict the status of the surrounding environment and the packaged food. Saenjaiban et al. [99] developed a TTI by incorporating silver nanoparticles and polydiacetylene into carboxymethyl cellulose films. These films demonstrated a noticeable color change from purplish-blue to reddish-purple in response to temperature and time. This color change is attributed to the increased surface area of polydiacetylene and the thermal conductivity of the silver nanoparticles.

#### 6.2.2. Freshness Indicators

Freshness indicators provide information on the quality of food in real-time by detecting the specific volatile compounds and pH of the packaged food. pH-sensitive colorimetric indicators like anthocyanin, alizarin, betaines, chlorophyll, and curcumin, and indicators that are sensitive to compounds like hydrogen sulfide, amines, ammonia, carbon dioxide, and ethylene are often used as freshness indicators in bionanocomposites for food packaging applications [100]. Khezerlou et al. [101] prepared two different freshness indicators based on gelatin/chitin nanofibers combined with saffron petal and barberry anthocyanins. Both indicators demonstrated noticeable color transitions across a range of pH levels (1–14). When applied to monitor-packaged fish, the saffron petal-based indicator exhibited a color shift from violet to green, whereas the one containing barberry anthocyanins changed from pink to yellow, indicating spoilage. Similarly, several researchers have explored the quality-monitoring ability of bionanocomposites prepared using anthocyanins from various sources [102,103]. Zhao et al. [104] incorporated nanolignin into cellulose nanofibrils and prepared a bionanocomposite film to monitor the freshness of food. They used fluorescein isothiocyanate as an indicator to detect biogenic amines, which are produced during food spoilage. The developed film displayed high sensitivity to biogenic amines, with a detection limit of as low as 1.83 ppm. They also developed a smartphone-compatible detection platform, enabling the real-time monitoring of food quality through accessible technology. Similarly, Ezati et al. [105] utilized resazurin as a freshness indicator due to its ability to undergo an oxidation-reduction reaction and produce a fluorescent pink-colored derivative called resorufin. They prepared a cellulose nanofiber-based film containing resazurin-modified carbon dots, which exhibited a color transition from yellow to brownish-red in response to alkaline pH and ammonia.

Silver nanoparticles have also been integrated into intelligent packaging systems due to their ability to detect spoilage gasses. In a study by Kwon and Ko [106], a composite film made from cellulose nanocrystals and silver nanoparticles was demonstrated to be able to indicate chicken breast quality. The initial color of the film ranged from yellowish to dark wine-red, depending on the concentration of silver nanoparticles. Upon exposure to hydrogen sulfide, the film gradually transitioned from its original color to colorless, and eventually to a metallic gray. This color change occurred due to alterations in the surface plasmon resonance of the silver nanoparticles, triggered by their interaction with hydrogen sulfide, making the film an effective visual indicator of food spoilage.

These advancements highlight the effectiveness of bionanocomposites in developing TTIs and freshness indicators for intelligent packaging. By providing real-time data on temperature exposure and spoilage detection, these systems significantly enhance food safety, traceability, and waste reduction. In addition, these systems allow for the better monitoring of food throughout the supply chain, ensuring customer satisfaction and improved inventory management, and reducing product losses.

### 6.3. Dual-Function Packaging

Advancements in bionanocomposites have paved the way for the development of multifunctional packaging systems that combine both active and intelligent components that enhance and monitor food quality. For instance, Sadi and Ferfera-Harrar [107] developed carboxymethyl cellulose/gelatin-based bionanocomposites containing montmorillonite as a nanofiller to improve the mechanical properties, anthocyanins as a colorimetric indicator to monitor pH changes, and *pistacia* leaf extract as an antimicrobial and antioxidant agent. These films not only enhance the quality and prolong the freshness of food but also provide real-time data on the quality of the food.

In some instances, the indicators used in smart packaging systems can also serve dual purposes by exhibiting active functionalities. For instance, barberry anthocyanin combined with methylcellulose/chitosan nanofibers not only indicate the freshness of packaged food by exhibiting color changes at different pH buffers and responding to different ammonia vapors, but also exhibit antioxidant activity [103]. In a similar study involving the development of PVA and high-amylose-starch films, [108] incorporated montmorillonite to enhance the physical, thermal, and permeable properties and anthocyanin to improve the antioxidant, antibacterial, and pH-response characteristics. The researchers also demonstrated its potential use as an active and intelligent packaging for chicken. Amaregouda et al. [109] prepared a chitosan and PVA-based film and demonstrated that the addition of anthocyanin–zinc oxide nanoparticles improved the mechanical, water-vapor-permeable, antioxidant, and antimicrobial properties of the chitosan/PVA films. In addition, they also demonstrated that the film could help monitor the freshness of packaged fish by changing from colorless to yellow. These multifunctional approaches help address the growing demand for smarter and more sustainable food packaging.

### 6.4. Packaging Material for Selected Food Commodities

Before considering the bionanocomposites used for packing a food product, it is crucial to evaluate the compatibility of the packaging material and the food. For instance, incorporating zinc oxide nanoparticles into kappa carrageenan bionanocomposites enhances their solubility, which is beneficial for edible films and products subjected to heating [110]. However, this nanocomposite is not suitable for the packaging of high-moisture foods like fresh fruits and vegetables, as the moisture content in the product can deteriorate the packaging material and expose the products to the outside environment. Moreover, the hydrophilic nature of the films tends to increase the water vapor permeability, another disadvantage for their use in high-moisture food packaging applications. Similarly, López et al. [111] packed cherry tomatoes in pure thermoplastic starch and thermoplastic starch incorporated with 5% talc powder and observed that tomatoes packed with bionanocomposites were inflated. Although the bionanocomposites demonstrated excellent seal and barrier properties, the inflated package would not be appealing on a market shelf. Hence, research is carried out to analyze the applicability of the nanocomposite for selected food commodities. Table 3 provides a detailed overview on the effect of bionanocomposites on selected food commodities.

The compatibility of active ingredients in packaging materials with food is also crucial. For instance, volatile sulfur compounds like hydrogen sulfide, and methanethiol, dimethyl sulfide, butanethiol, and methionol, found in foods like ham, peanuts, and par-baked buns, can result in the inactivation of palladium-based oxygen scavengers [112].

**Table 3:** Effect of bionanocomposites on selected food commodities.

Matrix	Reinforcements	Other Additional Materials	Food Material	Analyzed Properties of Food Material	Storage Period	Research Findings	References
Agar (obtained from *Gracilaria vermiculophylla*)	Zinc oxide nanoparticles	-	Smoked salmon	Microbial analysis, lipid oxidation (peroxide value and Thiobarbituric Acid Reactive Substances (TBARS))	4 °C; 8 days	Samples with 3% reinforcement had lower lipid oxidation and higher antimicrobial activity against *Listeria monocytogenes* and *Salmonella typhimurium*	[113]
Bovine skin gelatin	Zinc oxide nanorods	Clove essential oil	Shrimp	Microbial analysis	4 °C; 20 days	Bionanocomposites with 50% essential oil and 2% reinforcement exhibited maximum antimicrobial activity against *Listeria monocytogenes* and *Salmonella typhimurium* in shrimp.	[84]
Chitosan	Nanosized titanium dioxide	-	Cherry tomatoes	Firmness, weight loss, color, total soluble content, lycopene content, ascorbic acid, concentration of ethylene, carbon dioxide	20 °C; 14 days	Films exhibited ethylene photodegradation under ultraviolet light, delaying tomato-ripening and extending shelf life.	[85]
Chitosan	Cellulose nanocrystals	Carvacrol	Banana and Mango	Morphological changes, weight loss, hardness	25 °C; 7 days	Bionanocomposite-coated fruits exhibited superior qualities, with an increased hardness and reduced weight loss	[114]
Whey protein isolate	Starch nanocrystal	Jujube polysaccharide	Carrot	pH, weight loss, hardness, color, total psychotropic bacteria and total fungi, sensory properties	4 °C; 2 weeks	Bionanocomposite-wrapped carrots exhibited better stability and reduced microbial growth compared to PVC-wrapped carrots	[115]
Chitosan	Cloisite Na^+^ and Cloisite Ca^2+^	Rosemary and ginger essential oil	Poultry meat	Moisture, ash content, pH, total acidity, lipid oxidation, microbial analysis	5 ± 2 °C; 15 days	Nanoclay-reinforced nanocomposite reduced lipid oxidation by 50% and microbial contamination by 6–16% compared to pure chitosan film.Essential oil films significantly reduced oxidation.	[116]
Chitosan	Zinc oxide nanoparticles	Neem essential oil	Carrot	Microbial analysis, weight loss	-	Bionanocomposite-wrapped carrots had reduced weight loss and colony-forming units compared to those wrapped in commercial film or left uncovered on the 1st and 5th days	[89]
Chitosan	Sodium montmorillonite	Ginger essential oil	Poultry meat	Moisture, pH, titratable acidity, color, TBARS, Index, microbial analysis	5 ± 2 °C; 15 days	Meat samples covered with bionanocomposites showed extended shelf life with a reduced microbial count, no significant change in color orpH, and a slight increase in TBARS. The addition of essential oil to chitosan reduced lipid oxidation and microbial count, while in nanoclay-reinforced chitosan, it was less effective due to nanoclay blocking oil release.	[117]
Chitosan/Carboxymethyl cellulose	Zinc oxide nanoparticles	-	Egyptian soft white cheese	Rheological properties, color measurements, moisture, titratable acidity, pH, microbial analysis	7 °C; 30 days	Cheese packaged in bionanocomposites showed superior properties compared to traditional polystyrene package	[90]
Chitosaan/Guar gum	Zinc oxide nanoparticles	-	Ras cheese	pH, dry matter, soluble nitrogen, fat, ash, total and titratable acidity, microbial analysis	12 °C; 4 months	Cheese packed in bionanocomposites had a better shelf life, and improved chemical, microbial, and sensorial properties, compared to chitosan and guar gum coated and uncoated cheese	[91]
Pectin	Zinc oxide nanoparticles	-	Fresh poultry meat	Microbial analysis, pH, titratable acidity, moisture, total volatile basic nitrogen, color, TBARS	5 °C; 15 days	Pectin-based films reduced microbial growth on poultry meat, and the addition of zinc oxide nanoparticles further enhanced this effect. Bionanocomposite films greatly reduced the degradation of meat	[118]
Carboxymethyl cellulose/Okra mucilage	Zinc oxide nanoparticles	Nanostructured lipid carriers containing savory essential oil	Beef	Total volatile nitrogen, color, pH, TBARS, microbial analysis	4 °C; 12 days	Bionanocomposite-coated beef showed a significantly reduced microbial growth rate.	[119]
Gelatin	Nanocrystalline cellulose	Nanopropolis	Strawberry	Water content, vitamin C, hardness, total dissolved solids, pH, shelf life	5, 10 and 30 °C; 21 days	At 5 and 10 °C, the bionanocomposite coating increased the shelf life of strawberries.	[120]
Fish skin gelatin	Zinc oxide nanoparticles	Ginger essential oil	Meat	Total volatile bases’ nitrogen, peroxide value, microbial analysis	4 °C; 9 days	Bionanocomposites with a higher concentration of ginger essential oil, when used to wrap meat samples, showed a significant reduction in lower total volatile bases’ nitrogen, lipid oxidation, and microbial count	[121]
Poly (lactic acid)	Cloisite Na^+^	-	Processed meat product	Lipid oxidation (hexanal, TBARS, p-anisdine)	5 °C; 90 days	Bionanocomposites reduced lipid oxidation and extended the shelf life of the packaged food.	[122]
Starch	Halloysite	Nisin	Minas Frescal Cheese	Antimicrobial analysis	4 ± 2 °C; 14 days	The nanocomposite with a higher clay content delayed nisin diffusion, reducing antimicrobial activity among microorganisms compared to the composite with same nisin content and lower nanoclay contents. However, nanocomposites with higher concentrations of nisin (6 g/ 100 g) showed a significant invrease in inactivation after 4 days, continuing through the 14-day study period	[86]

While synthetic polymers generally offer better mechanical and barrier properties compared to those of bionanocomposites, the presence of active ingredient in the bionanocomposites can help enhance the shelf life and quality of the food material. Kumar et al. (2018) [9] wrapped red grapes with plastic film, chitosan/gelatin film, and silver nanoparticle (0.05, 0.1%) incorporated chitosan/ gelatin bionanocomposites and evaluated the shelf life of grapes at 37 °C for 14 days. The plastic film wrapped grapes were found to be sticky with molds and rotten smell; while bionanocomposites wrapped red grapes were fresh for the tested period. Similarly, laponite immobilized silver nanoparticles incorporated chitosan films were able to keep the litchi fruits fresher than the commercial polyethylene-based cling wraps were used [123]. Arfat et al. [124] prepared bionanocomposites with fish protein isolate/fish skin gelatin (FPI/FSG), zinc oxide nanoparticles (ZnO), and basil leaf essential oil (BEO). The bionanocomposite (FPI/FSG/ZnO/BEO) was used to wrap sea bass slices and the effect on the wrapped samples was analyzed after 12 days at 4 °C, focusing on pH, peroxide value, total volatile base, Thiobarbituric Acid Reactive Substances (TBARS) value, antimicrobial properties, and sensory analyses. Moreover, the bionanocomposite-wrapped samples were compared with those wrapped with FPI/FSG/BEO, FPI/FSG/ZnO, FPI/FSG, and polypropylene film, and those exposed to ambient conditions (unwrapped). The study found that the developed bionanocomposite could extend the shelf-life of sea bass slices by up to 12 days when compared to the 4, 5, 6, and 10 days obtained with unwrapped, polypropylene, FPI/FSG, and FPI/FSG/ZnO, respectively [124]. Similar results were reported by Sasidharan et al. [125], who compared poultry meat wrapped with a chitosan-based nanocomposite prepared with ZnO nanoparticles (prepared through green and chemical synthesis), chitosan, PVA, and unwrapped meat. They concluded that bionanocomposite containing green synthesized ZnO nanoparticles showed a greater improvement in maintaining the quality of meat than the chemogenic nanocomposite, chitosan, PVA, and unwrapped meat.

Przybyszewska et al. [118] wrapped poultry meat with a pectin- and ZnO-based bionanocomposite. After 15 days of refrigerated storage, the total zinc migration into the meat was found to be between 30.3 and 38.4 mg of zinc/kg of meat, whereas the fresh meat on day 0 contained 11.2 mg of zinc/kg. Although the use of ZnO-based bionanocomposites presents some risk regarding zinc migration, the actual intake after consuming a medium portion of meat (about 100 g) would be around 3.0 to 3.8 mg of zinc. This amount represents only 12–15% of the recommended upper intake limit of zinc per day, which is 25 mg/person. Similarly, Sasidharan et al. [125] also found 50 to 75 mg of zinc/ kg in poultry meat wrapped with a bionanocomposite prepared with chitosan and green synthesized ZnO, whereas, about 66 to 103 mg of zinc/kg of meat was observed on poultry meat wrapped with a chemogenic nanocomposite (containing chitosan and chemically synthesized ZnO). Thus, the amount of zinc that migrates into the food material also depends on the concentration of zinc present in the bionanocomposite, the method of preparation, the characteristics of the food material, and environmental conditions. Based on the results of these studies, it can be concluded that the use of a ZnO-based bionanocomposite as the primary packaging for poultry may not pose a significant risk in terms of zinc intake. Similar migration studies must be performed for all bionanocomposites intended for food-contact applications.

## 7. Nanomaterial Migration and Environmental Concerns

Despite bionanocomposites presenting a boon to food packaging, potential risks arise from the migration of nanomaterials into food. However, a complete understanding of the migration of nanomaterials to food and their effect on human health is lacking. This might be due to the difficulties in characterizing nanomaterials in complex food matrices and the lack of toxicological data. Moreover, the migration of nanomaterials depends on many factors, including the storage time and temperature, material properties, position of the migrant in the material, concentration gradient, interaction between the migrant and the material, and the characteristics of the food, such as pH. For instance, Metak et al. [126] reported that the migration of silver from nano-silver-coated films was the highest in orange juice compared to other food materials, such as bread, apple, cheese, milk powder, carrot, ground beef, butter, and water. This implies that a higher concentration of silver migrates in the presence of acidic foods. Also, the risk that is involved depends not only on the rate of migration and the toxicity of the nanomaterials, but also on how much of the food is consumed.

Effects on eco-toxicological information are also a major factor to consider for bionanocomposites that are disposed into the eco-system after usage. The improper disposal of bionanocomposites can lead to the release of nanomaterials into the environment, potentially causing toxicity in both soil and water ecosystems. These materials may accumulate over time, disrupting terrestrial and aquatic life. A recent study on the use of soy protein isolate–cellulose nanocrystal coatings for fruits like avocado has shown that the production process results in a carbon footprint of 0.33 kg carbon dioxide eq/kg of solution. This is higher than water-based wax coatings but lower than solvent-based alternatives [127]. This finding emphasizes the importance of balancing economic and environmental factors when scaling up the potential of such coatings for commercial use. Continuous casting is also being explored as a more efficient method for the production of gelatin–cellulose nanocrystal bionanocomposites. This process significantly enhances productivity, achieving a rate that is over 1000 times greater than traditional bench casting [128]. These innovations are critical for overcoming barriers to the commercial viability of bionanocomposites in flexible food packaging, where cost-effective and high-performance materials are essential for wider industry adoption.

Although EC regulations (No. 1935/2004) [129] exist for food-contact materials, active packaging materials are exempted from this regulation and are regulated by EC No. 450/2009 [130]. This regulation defines the materials that can be used for active packaging, as well as the amount of materials that can be released into the food material and/or headspace [131]. However, no standard limits or lists of nanomaterials that can be used for food contact application are explicitly provided. Only the overall migration of the nanomaterials can be assessed to ensure that the nanomaterials used for food-contact and/or packaging applications comply with the regulations. Although studies have proven that the migration of silicate layered nanoclays is insignificant/nil, regulations governing their use in the food industry are not available. Unlike their larger counterparts, nanoparticles are not covered by prior regulations. Materials not listed in the European Union index are restricted to non-food-contact uses. Nanomaterials must undergo stringent individual risk assessments, similar to the processes applied to mutagens and carcinogens. Furthermore, the lack of universally accepted protocols and standardized techniques for detecting nanomaterials in complex food matrices adds to the challenge of ensuring safety in food packaging. Standardized testing methods are crucial to accurately assess nanomaterial migration and its potential health risks.

## 8. Conclusions

Compared to polymeric nanocomposites, bionanocomposites hold significant potential for food packaging applications due to their biocompatibility, food-safe properties, and eco-friendly disposal options. While the research exploring the use of various combinations of biopolymer matrices and nanomaterials for the packaging of different food products is extensive, the commercial application of bionanocomposites remains limited. According to data from the Web of Science, out of over 17,000 articles published on bionanocomposites, at least 2000 are directly related to packaging or coating applications; however, their market penetration remains minimal. This limitation is likely due to challenges in scaling up production, including complex processing techniques and high costs. Additionally, achieving a consistent performance in terms of mechanical strength, barrier properties, and microbial activity while maintaining the environmental benefits has proven difficult. Regulatory challenges, as well as competition from cost-effective traditional packaging materials, further limit their use in the market. Addressing these issues could unlock the full potential of bionanocomposites as sustainable, advanced packaging solutions, particularly as consumer demand for biodegradable and eco-friendly materials continues to rise.

Future research should focus on addressing key challenges in the development and commercialization of bionanocomposites for food packaging. Priorities include in-depth studies on the migration of nanomaterials into food, with an emphasis on understanding their long-term health impacts and eco-toxicological effects after disposal. Developing standardized methods to assess nanomaterial migration and toxicity is essential. Lifecycle assessments could help identify the environmental trade-offs associated with bionanocomposites, as they evaluate resource use, energy consumption, and waste generation during production, transportation, usage, and disposal. Moreover, scalable production methods like continuous casting require optimization to reduce costs and improve the mechanical, barrier, and antimicrobial properties of bionanocomposites. Thus, updating regulatory guidelines, establishing industry standards, and investing in sustainable production technologies could pave the way for the broader use of bionanocomposites in food packaging.

## Data Availability

No new data were created or analyzed in this study. Data sharing is not applicable to this article.
